# Changes in intracellular folate metabolism during high-dose methotrexate and Leucovorin rescue therapy in children with acute lymphoblastic leukemia

**DOI:** 10.1371/journal.pone.0221591

**Published:** 2019-09-17

**Authors:** Natanja Oosterom, Robert de Jonge, Desiree E. C. Smith, Rob Pieters, Wim J. E. Tissing, Marta Fiocco, Bertrand D. van Zelst, Marry M. van den Heuvel-Eibrink, Sandra G. Heil

**Affiliations:** 1 Princess Máxima Center for Pediatric Oncology, Utrecht, The Netherlands; 2 Erasmus MC, University Medical Center Rotterdam, Department of Clinical Chemistry, Rotterdam, The Netherlands; 3 VU Medical Center, Department of Clinical Chemistry, Amsterdam, The Netherlands; 4 Academic Medical Center, Department of Clinical Chemistry, Amsterdam, The Netherlands; 5 Department of Pediatric Oncology, University of Groningen, Beatrix Children’s Hospital, University Medical Center Groningen, Groningen, The Netherlands; 6 Mathematical Institute, Leiden University, Leiden, The Netherlands; 7 Leiden University Medical Center, Department of Biomedical Data Sciences, Leiden, The Netherlands; University of Texas at Austin, UNITED STATES

## Abstract

**Background:**

Methotrexate (MTX) is an important anti-folate agent in pediatric acute lymphoblastic leukemia (ALL) treatment. Folinic acid rescue therapy (Leucovorin) is administered after MTX to reduce toxicity. Previous studies hypothesized that Leucovorin could ‘rescue’ both normal healthy cells and leukemic blasts from cell death. We assessed whether Leucovorin is able to restore red blood cell folate levels after MTX.

**Methods:**

We prospectively determined erythrocyte folate levels (5-methyltetrahydrofolate (THF) and non-methyl THF) and serum folate levels in 67 children with ALL before start (T0) and after stop (T1) of HD-MTX and Leucovorin courses.

**Results:**

Erythrocyte folate levels increased between T0 and T1 (mean ± SD: 416.7 ± 145.5 nmol/L and 641.2 ± 196.3 nmol/L respectively, p<0.001). This was due to an increase in 5-methyl THF levels (mean increase: 217.7 ± 209.5 nmol/L, p<0.001), whereas non-methyl THF levels did not change (median increase: 0.6 nmol/L [-9.9–11.1], p = 0.676). Serum folate levels increased between T0 and T1 (median increase: 29.2 nmol/L [32.9–74.0], p<0.001). Results were not significantly affected by age, sex, ALL immunophenotype and MTHFR c.677C>T genotype.

**Conclusion:**

Intracellular folate levels accumulate after HD-MTX and Leucovorin therapy in children with ALL, suggesting that Leucovorin restores the intracellular folate pool. Future studies are necessary to assess concomitant lower uptake of MTX.

## Introduction

**Fig 1:**
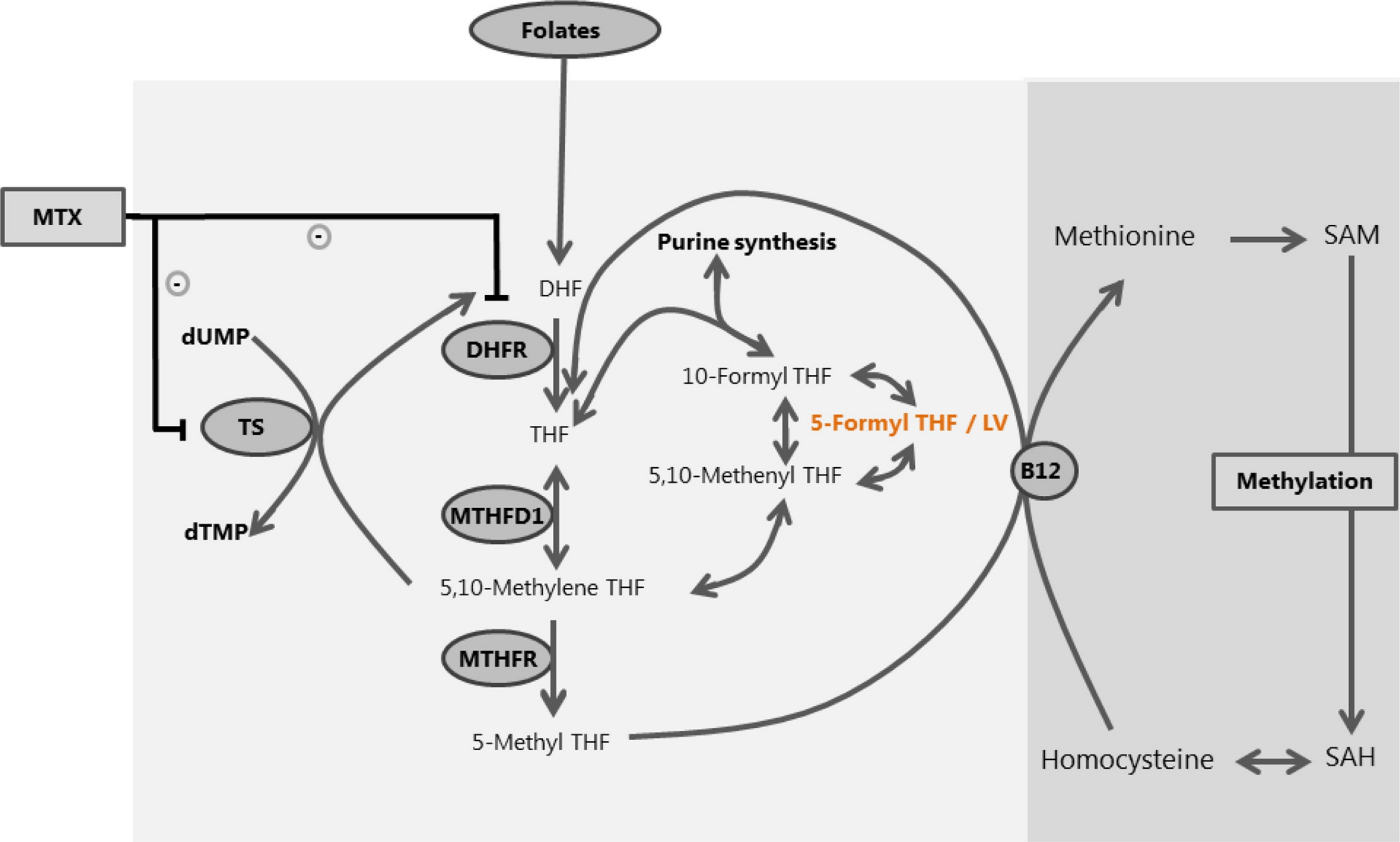
Folate pathway. Overview of the folate pathway with separate folate isoforms and converting enzymes. Leucovorin (5-formyl THF) rescue therapy bypasses the action of DHFR. SAM: S-adenosylmethionine; SAH: S-adenosylhomocysteine; DHF: dihydrofolate; THF: tetrahydrofolate; TS: thymidylate synthase; DHFR: dihydrofolate reductase; MTHFD1: methylenetetrahydrofolate dehydrogenase 1; MTHFR: methylenetetrahydrofolate reductase; MTX: methotrexate; LV: Leucovorin.

High-dose methotrexate (HD-MTX) is an important component of pediatric acute lymphoblastic leukemia (ALL) treatment. [1–3] Methotrexate is a cytotoxic agent that depletes intracellular reduced folate levels by inhibiting the enzymes Dihydrofolate Reductase (DHFR) and Thymidylate Synthase (TYMS) (Fig 1). [4–6] MTX thereby inhibits DNA- and RNA synthesis. [4–6] After HD-MTX infusions, folinic acid rescue therapy (Leucovorin) is administered to reduce toxic side effects of therapy. [7]

Previous studies showed that red blood cell folate levels have been low in patients treated with only MTX without Leucovorin. [8–10] Leucovorin and MTX are structural analogues and could therefore compete for cellular transport-, polyglutamylation-, and enzyme-binding (DHFR/TS) mechanisms. [11, 12] As a reduced folate, Leucovorin could also bypass the enzyme DHFR during consecutive HD-MTX and LV courses, thereby restoring purine- and pyrimidine- synthesis. It has been hypothesized that Leucovorin restores cellular folate levels after MTX (Fig 1). This phenomenon has been referred to as the folate ‘overrescue’ principle, where not only healthy cells, but also tumor cells are ‘rescued’ from cell death due to Leucovorin after HD-MTX.

Both in pediatric ALL studies [13–16] as in rheumathoid arthritis studies [17, 18] results suggested that Leucovorin rescue therapy decreases toxicity rates, but showed an increased risk of relapse in ALL and decreased treatment efficacy in RA. In contrast, others have successfully introduced early LV rescue in individualized doses after MTX in treatment protocols with high event-free survival rates. [19–21] In addition, previous studies failed to detect correlations between red blood cell (RBC) MTX and folate levels measured at end of intensification therapy and throughout maintenance therapy in relation to complete continuous remission and event-free survival rates. [8, 21] These contradictory study results emphasize the difficulties still encountered in understanding the impact in Leucovorin and MTX on pediatric ALL treatment outcome. This is undesirable as both treatment efficacy and toxicity are affected by the possible competition between MTX and Leucovorin. Until now, no studies have assessed the effect of 4x consecutive HD-MTX (5000 mg/m^2^/dose) and Leucovorin on RBC folate levels.

Different folate vitamers are involved in intracellular folate metabolism. Intracellular folate levels can be divided into two pools: the 1) 5-methyl tetrahydrofolate (THF) pool serving the methylation cycle and the 2) non-methyl THF pool serving the DNA/RNA biosynthesis cycle (Fig 1). In plasma, the main folate isoform that accumulates after Leucovorin (folinic acid; 5-formyl THF) infusion is 5-methyl tetrahydrofolate (5-methyl THF) (Fig 1). [22–24] In mature erythrocytes, which are regarded as the best intracellular reflection of the whole-body folate status over the past three months, 5-methyl THF is the largest pool. [25] Previous studies showed that after intravenous and oral administration of Leucovorin, 5-formylTHF, THF and 5,10-methylTHF had a short T_1/2_ of 30 minutes after administration, after which 5-methylTHF accumulated. [24, 26–28] In addition, homocysteine and vitamin B12 are important biomarkers of intracellular one-carbon metabolism (Fig 1). Homocysteine reflects changes in the one-carbon metabolism due to disturbances of folate- and vitamin B12 levels and increases in response to MTX therapy. [29, 30] Vitamin B12 is involved in one-carbon metabolism as co-enzyme facilitating the re-methylation of homocysteine into methionine (Fig 1). [31]

This study aims to determine whether serum and intracellular folate levels accumulate during four consecutive HD-MTX and Leucovorin courses in children with ALL. In addition, we studied homocysteine and vitamin B12 levels during HD-MTX and Leucovorin courses.

## Results

### Patient characteristics

**Fig 2:**
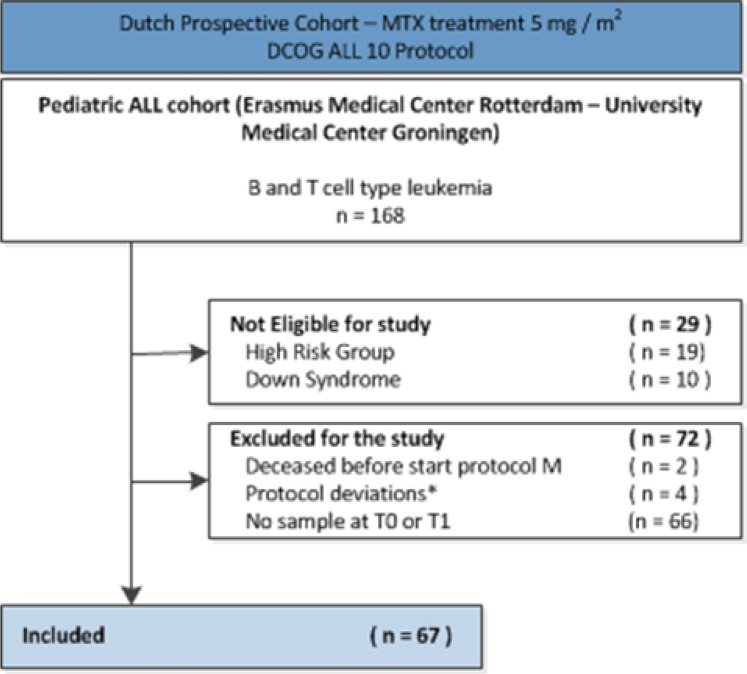
Flowchart patient inclusion. Abbreviations: ALL, acute lymphoblastic leukemia; HD-MTX, high-dose methotrexate; DCOG, Dutch Childhood Oncology Group; SNP, single-nucleotide polymorphism; n = number of patients; * 1 patient had neurological damage before start HD-MTX treatment, 1 patients was transferred to another hospital, 1 patient had an adjusted protocol due to a SPINKS mutation and 1 patient was initially treated otherwise due to another diagnosis.

In total, 67 children with ALL treated according to the Dutch Childhood Oncology Group ALL-10 protocol (S1 Fig) were included in this study (Fig 2). The median age at diagnosis was 5.4 years (range 1.4–17.5). Thirty patients were female (45%), 48 patients were treated in the medium risk group (72%) and 58 patients had a B-lineage ALL (87%). Thirty-two (48%) patients were carriers of the MTHFR 677 CC genotype, 24 (36%) patients of the MTHFR 677 CT genotype, 10 (15%) patients of the MTHFR 677 TT genotype and in 1 (1%) patient the genotype was missing.

### Laboratory measurements before start (T0) and after stop (T1) of HD-MTX treatment

**Fig 3:**
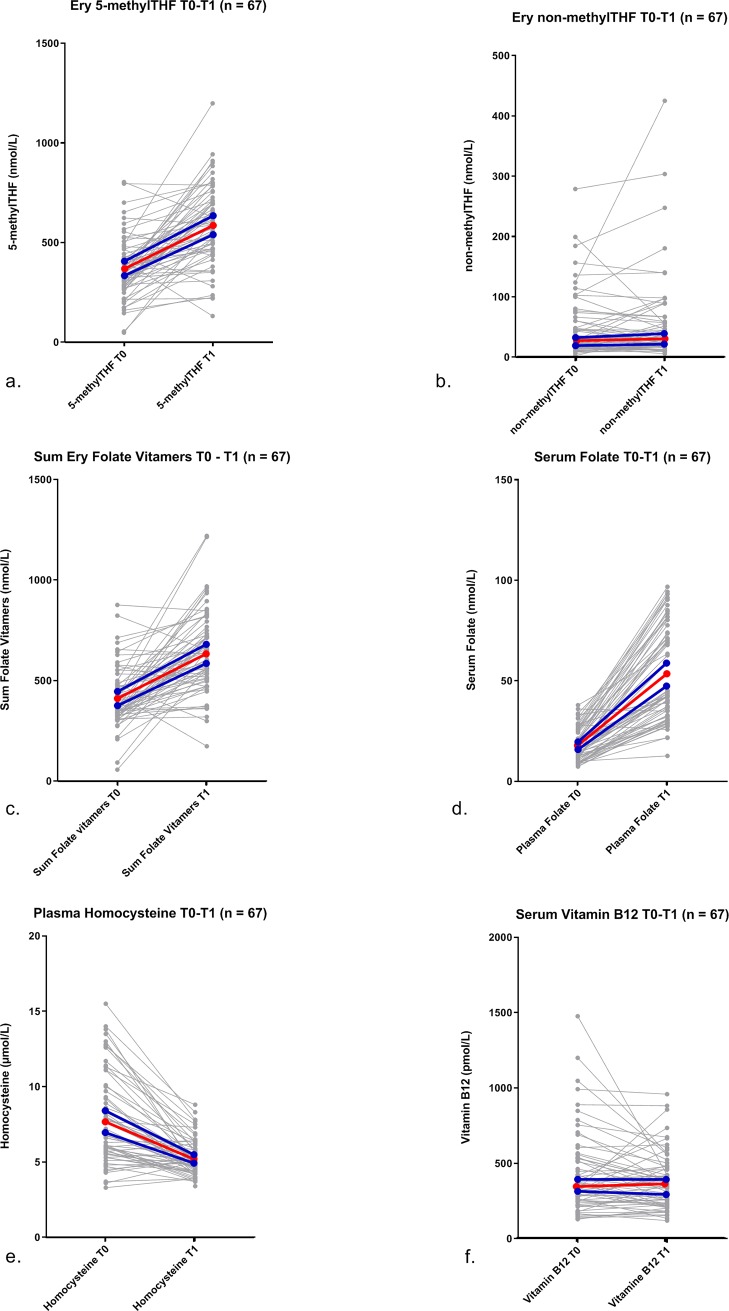
One carbon metabolism metabolite levels before (T0) and after (T1) high-dose MTX therapy. One carbon metabolism metabolite levels before (T0) and after (T1) of high-dose MTX therapy: erythrocyte 5-methyl THF (a, p<0.0001), erythrocyte non-methyl THF (b, p = 0.676) sum erythrocyte folate isoforms (c, p<0.0001), serum folate (d, p <0.0001), plasma homocysteine (d, p<0.0001), serum vitamin B12 (e, p = 0.134). The red lines depict the median levels and the blue lines depict the 95% confidence interval.

Erythrocyte folate increased significantly between T0 and T1 (mean 224.4 ± 220.7 nmol/L) (Table 1 + Fig 3). This increase was due to an increase in erythrocyte 5-methyl THF levels (mean 217.7 ± 209.5 nmol/L), whereas erythrocyte non-methyl THF levels remained stable (median 0.6 nmol/L [-9.9–11.1]) throughout treatment. Serum folate levels increased significantly between T0 and T1 (median 29.2 nmol/L [19.3–57.4]), plasma homocysteine levels decreased significantly (-2.5 ± 2.5 μmol/L), while serum vitamin B12 levels did not change (median -4.9 pmol/L [-131.0–40.9], p = 0.134) (Table 1 + Fig 3). Due to the presence of extreme outliers for the Delta T1 –T0, a sensitivity analysis with and without outliers was performed. Results did not change after the sensitivity analysis (data not shown). No correlation between erythrocyte folate levels at T0/T1 and erythrocyte MTX-PG_1-5_ at T1 existed (data not shown).

**Table 1:** One-carbon metabolism metabolite levels at T0 and T1 (n = 67).

	T0 (before start MTX)	T1 (after stop MTX)	Delta T1 –T0	p-value
**Ery 5-methyl THF (nmol/L), mean ± SD**	370.2 ± 149.3	587.8 ± 194.6	217.7 ± 209.5	<0.0001*
**Ery Non-methyl THF (nmol/L), median (IQR)**	27.0 (17.1–48.4)	30.4 (18.6–56.3)	0.6 (-9.9–11.1)	0.676
**Ery Sum Folate Level^a^ (nmol/L), mean ± SD**	416.7 ± 145.5	641.2 ± 196.3	224.4 ± 220.7	<0.0001*
**Plasma Homocysteine (μmol/L), mean ± SD^b^**	7.8 ± 2.9	5.3 ± 1.2	-2.5 ± 2.5	<0.0001*
**Serum Vitamin B12 (pmol/L), median (IQR)**	345.6 (255.9–531.4)	363.2 (233.8–465.3)	-4.9 (-131.0–40.9)	0.134
**Serum Folate (nmol/L), median (IQR)**	16.4 (11.5–24.3)	42.8 (32.9–74.0)	29.2 (19.3–57.4)	<0.0001*

^a^ sum 5-methylTHF + non-methylTHF

^b^ In n = 2 patients homocysteine levels were missing.

*p-value < 0.05. Univariate analysis of changes of folate metabolism markers between T0 and T1 using a paired T test (normally distributed data; mean ± Standard Deviation (SD)) or a Wilcoxon Singed Rank Test (skewed distributed data; median, Interquartile Range (IQR)).

Reference values in healthy population

Ery 5-methylTHF (nmol/L) median (range) 427.3 (92.5–1085.8)^c^

Ery non-methylTHF (nmol/L) median (range) 4.1 (0–786.2)^c^

Ery sum folate level (nmol/) median (range) 440.9 (170.3–1164.4)^c^

Plasma Homocysteine (μmol/L) reference interval 5.0–15.0^d^

Serum Vitamin B12 (pmol/L) reference interval 145–637^d^

Serum folate (nmol/L) reference interval 5–40^d^^c^

^c^https://www.sciencedirect.com/science/article/pii/S0955286307000149?via%3Dihub

^d^According to reference values of our laboratory

### Covariates

In univariable analysis, ALL immunophenotype was significantly associated with the Delta T1 –T0 erythrocyte 5-methyl THF levels and the Delta T1 –T0 erythrocyte sum folate levels and age was significantly associated with the Delta T1 –T0 plasma homocysteine levels (data not shown). However, none of these confounders significantly affected the Delta T1 –T0 in a linear regression model (Table 2).

**Table 2:** Estimated linear regression coefficients with 95% confidence intervals.

		β	95% CI
	**Ery 5-methyl THF (nmol/L)**		
**Model 1**	Delta Ery 5-methyl THF (nmol/L) T1 –T0Ery 5-methyl THF (nmol/L) T0	-0.63	(-0.95 –-0.32)
**Model 2**	Delta Ery 5-methyl THF (nmol/L) T1 –T0 Ery 5-methyl THF (nmol/L) T0 ALL immunophenotype	-0.59^#^	(-0.92 –-0.26)
	**Ery Sum Folate Vitamers (nmol/L)**		
**Model 1**	Delta Ery Sum Folate Vitamers (nmol/L) T1 –T0 Ery Sum Folate Vitamers (nmol/L) T0	-0.75	(-1.07 –-0.41)
**Model 2**	Delta Ery Sum Folate Vitamers (nmol/L) T1 –T0 Ery Sum Folate Vitamers (nmol/L) T0ALL immunophenotype	-0.70^#^	(-1.04 –-0.36)
	**Plasma Homocysteine (μmol/L)**		
**Model 1**	Delta Plasma Homocysteine (μmol/L) T1 –T0 Plasma Homocysteine (μmol/L) T0	-0.77	(-0.85 –-0.69)
**Model 2**	Delta Plasma Homocysteine (μmol/L) T1 –T0 Plasma Homocysteine (μmol/L) T0Age	-0.83^#^	(-0.91 –-0.74)

Possible confounders that were both significantly associated with T0 and delta T1-T0 were entered in model 2 to see whether the β changed >10%; ALL immunophenotype was added to model 2 for Ery 5-methyl THF and Ery Sum Folate vitamers and age for Plasma Homocysteine. β = regression coefficient, SE = Standard Error of β, 95% CI = 95% Confidence Interval of β

^#^Change in β was <10%.

### One-carbon metabolism markers in relation to toxicity

Changes in erythrocyte- and plasma folate, plasma homocysteine and plasma vitamin B12 levels between T0 and T1 were not significantly associated to the development of MTX-induced oral mucositis NCI≥ grade 3 (Table 3).

**Table 3:** Delta one-carbon metabolism levels in relation to MTX-induced oral mucositis (n = 67).

Delta T1 –T0	n	(%)	nmol/L		p-value	p-value corrected^a^	OR (95% CI)	
**5-methyl THF, median (IQR)**								
Mucositis–no	53	(79)	205.21	(103.9–414.0)	0.157	0.215	0.997	(0.993–1.002)
Mucositis—yes	14	(21)	40.6	(-9.1–287.9)				
**Non-methyl THF, median (IQR)**								
Mucositis–no	53	(79)	0.6	(-10.9–10.5)	0.974	0.864	0.999	(0.987–1.011)
Mucositis—yes	14	(21)	-1.1	(-11.1–18.6)				
**Sum Folate^b^, median (IQR)**								
Mucositis–no	53	(79)	201.2	(96.9–411.0)	0.176	0.402	0.999	(0.995–1.002)
Mucositis—yes	14	(21)	129.1	(34.4–297.5)				
**Plasma Homocysteine^c^, median (IQR)**								
Mucositis–no	51	(78)	-2.1	(-3.6 - -0.6)	0.737	0.085	0.509	(0.236–1.098)
Mucositis–yes	14	(22)	-1.6	(-5.1 - -0.8)				
**Plasma Vitamin B12, median (IQR)**								
Mucositis–no	53	(79)	-4.9	(-131.8–51.6)	0.554	0.866	1.000	(0.995–1.004)
Mucositis–yes	14	(21)	2.2	(-114.6–26.6)				
**Plasma Folic acid (nmol/L), median (IQR)**								
Mucositis–no	53	(79)	29.7	(21.5–57.4)	0.262	0.241	0.981	(0.951–1.013)
Mucositis–yes	14	(21)	23.4	(11.7–53.1)				

^a^ Delta variables corrected for value at T0, age and gender; 5-methylTHF / non-methylTHF pool corrected for age, gender and MTHFR C667T genotype

^b^ sum 5-methylTHF + non-methylTHF.

^c^ In n = 2 patients homocysteine levels were missing.

## Discussion

In this study we assessed intracellular folate levels throughout HD-MTX and Leucovorin therapy in children with ALL. We showed that intracellular 5-methyl THF levels increased during high-dose MTX and Leucovorin therapy, suggesting that Leucovorin is able to restore the intracellular folate pool.

In this study, we observed an increase in 5-methyl THF pool, whereas the non-methyl THF pool did not change. It is likely that throughout the course of four HD-MTX and Leucovorin courses in 57 days the non-methyl THF pool is largely reduced to 5-methyl THF. The increase of folate levels observed in patients receiving MTX and Leucovorin is likely due to Leucovorin, as previous studies showed low red blood cell folate levels in patients treated with MTX monotherapy. [8–10] Our findings are in line with findings of Holmboe *et* al (2012), who showed a similar increase in erythrocyte folate levels in osteosarcoma patients receiving HD-MTX and LV. [32] However, as we could not compare our data to patients that received a similar treatment protocol with HD-MTX without Leucovorin, we could not exclude effects of other co-medication such as 6-Mercaptopurine or nutritional factors.

In this study, we did not find an association between the change in folate metabolism markers and the development of oral mucositis NCI grade≥3 throughout protocol M. However, in this study design we only had folate metabolism markers at start and at the end of protocol M and we could have missed effects throughout the four different HD-MTX courses.

Homocysteine is a sensitive biomarker of one-carbon / folate metabolism (Fig 1), that was shown to be increased in plasma in response to MTX administration. [16, 33] This initial increase in homocysteine levels was reversed after start of Leucovorin. [16] In line with these previous results, plasma homocysteine levels in our study decreased after high-dose MTX and Leucovorin therapy confirming that the intracellular folate pool was restored.

In recent years concerns have been raised about the possible adverse effects of excessive folate intake in the field of folic acid food fortification due to the ‘methylfolate trap’. [34] This ‘methylfolate trap’ is based on the fact that the conversion of 5,10-methylene THF to 5-methyl THF is irreversible (Fig 1). To maintain an intact folate cycle, 5-methyl THF can only be converted to THF by the vitamin B12-dependent re-methylation of homocysteine to methionine. If either folate levels are excessively high or if vitamin B12 levels are low, this could then lead to ‘trapping’ of 5-methyl THF with concomitant high levels of homocysteine and subsequently the impairment of essential intracellular methylation and DNA / RNA synthesis processes. [35, 36] In the setting of our study, patients received Leucovorin, which led to iatrogenic increased intracellular folate levels. However, as homocysteine levels decreased during therapy and vitamin B12 levels were within the normal range (>148 pmol/L), we did not find evidence of the ‘methylfolate trap’.

We have demonstrated that intracellular folate levels increase upon HD-MTX and Leucovorin treatment in pediatric ALL-patients. It is of interest to assess the magnitude of this increase in relation to intracellular folate levels found in healthy individuals. Compared with healthy adults the median sum of the intracellular folate vitamers at T1 are increased, but the majority of values are within the large normal range found in the healthy adult population (median 440.0 nmol/L, range 170.3–1164.4]). [25] The large interindividual variability in intracellular erythrocyte folate levels in our pediatric ALL patients are in line with the variability reported in the healthy population and might be partly explained by pre-analytical conditions, genetic variation in genes of folate transporters and metabolizing enzymes, such as the MTHFR c.677 C>T genotype, as well as differences in dietary folate intake. [25]

We showed that intracellular folate levels before start of the first HD-MTX (T0) course and after stop of four HD-MTX (T1) courses were not correlated to intracellular MTX-PG_1-5_ after stop of four HD-MTX (T1) courses in this study. This needs to be assessed in more detail in future studies. Previous studies showed that side effects, such as oral mucositis, predominantly occur during the first out of four HD-MTX courses. [37] It would be of value to assess intracellular folate- and MTX levels more frequently throughout the treatment protocol as it could be that competition for cellular transport mechanisms exists more early in the treatment protocol.

In this study, we measured intracellular folate levels in erythrocytes. Erythrocytes are expected to be a reflection of intracellular folate status of other cells such as leucocytes, as erythroblasts take up folate and most of this pool is retained throughout its lifespan. However, it would be of scientific value to determine folate levels in nuclear cells such as leucocytes, as erythrocytes do not have a nucleus nor mitochondria and therefore no active formation of DNA- or RNA structures. [38]

Strengths of this study are the prospective collection of samples and the erythrocyte folate vitamer level measurements. A limitation is that we only measured folate levels before start of HD-MTX therapy and after stop of HD-MTX therapy, whereas in future studies it would be valuable to measure these levels more frequently throughout the treatment protocol.

In conclusion, intracellular folate levels accumulate during HD-MTX and Leucovorin therapy in children with ALL with a concomitant decrease in plasma homocysteine levels. These results support the hypothesis that Leucovorin restores the intracellular folate pool during treatment with MTX. Future studies that determine whether intracellular folate levels are associated with a decrease in intracellular MTX levels throughout the HD-MTX treatment protocol are of interest to assess whether competition between MTX and Leucovorin exists for cellular transport mechanisms.

## Methods

### Patient selection

Pediatric ALL patients (1–19 years) treated with HD-MTX courses according to the standard and medium risk arms of the Dutch Childhood Oncology Group ALL-10 protocol were eligible for this study. [39] We obtained written informed consent from patients and their parents before sample collection according to the Declaration of Helsinki. The study was approved by the Medical Ethical Committee Erasmus Medical Center (Erasmus MC, Rotterdam, The Netherlands, MEC-2005-358). Children were diagnosed with ALL in the period between 2004 and 2012 in two Dutch pediatric oncology centers (the Erasmus MC–Sophia Children’s Hospital in Rotterdam and the University Medical Center Groningen (UMCG)–Beatrix Children’s Hospital in Groningen). We studied patients prospectively during protocol M (4x 5g/m2 high-dose MTX courses). Detailed information on this protocol and cohort has been previously described. [37] In short, four HD-MTX infusions were administered every two weeks at a dose of 5000 mg/m2 in 24 hours. Each MTX administration was combined with intrathecal triple chemotherapy in a standard dose adjusted for age (8–12 mg MTX; 20–30 Cytosine Arabinoside; 8–12 mg Diadreson F aquosum). Folinic acid rescue therapy (15 mg/m^2^/dose) was administered at 42, 48 and 54 hours after the start of MTX administration. Standard supportive care guidelines included hyperhydration (2.5–3.0 L/m^2^/day) and urine alkalinization using sodium bicarbonate (pH between 7.0–8.0). In addition, protocol M included oral 6-mercaptopurine (25 mg/m^2^ daily for 56 days).

### Erythrocyte folate, serum folate, plasma homocysteine and serum vitamin B12 measurements

Red blood cell pellets, serum and EDTA plasma samples were collected from the patients before the start of protocol M (T0) and two weeks after discontinuation of protocol M (T1) for measurement of erythrocyte folate levels (S1 Fig). All samples were stored at − 80 °C and analyzed collectively. Erythrocyte folate levels (non-methyl tetrahydrofolate (non-methyl THF) pool; 5-methyl tetrahydrofolate (5-methyl THF) pool; folic acid pool) were measured using liquid chromatography-tandem mass spectrometry as previously described. [40] The non-methyl THF pool consists of the sum of THF, 5,10-methylene THF, 5,10-methenyl THF and 5- and 10-formyl THF (Fig 1). As folic acid concentrations were negligible (median 1.2; range 0.5–7.3 nmol/L), the sum of the total erythrocyte folate level was calculated by adding the non-methyl THF and 5-methyl THF pools. Serum folate levels and serum vitamin B12 levels were measured using an electrochemiluminescence immunoassay (Modular E170, Roche, Almere, The Netherlands). Plasma homocysteine levels were measured using liquid chromatography-tandem mass spectrometry as previously described. [41] Samples of erythrocyte MTX-polyglutamates (MTX-PG_1-5_) were only collected at T1 and were measured as previously described. [42]

### Statistical analysis

A Paired-Samples T-test (normally distributed variables; mean ± SD) and a Wilcoxon Signed Rank test (skewed variables; median [interquartile range]) were used to compare mean or median erythrocyte 5-methyl THF, erythrocyte non-methyl THF, erythrocyte sum folate, serum folate, plasma homocysteine and serum vitamin B12 levels at T0 and T1 in univariate analysis. A linear regression model was estimated to assess the effect of age at diagnosis, sex, ALL immunophenotype and MTHFR c.677C>T genotype on the absolute changes in these folate metabolism markers between T0 and T1 (Delta T1 –T0), while correcting for the levels at T0. A logistic regression model was estimated to assess the effect of the change in folate metabolism markers between T0 and T1 (Delta T1-T0) in relation to the development of MTX-induced oral mucositis NCI grade≥3, while correcting for age at diagnosis, sex, ALL immunophenotype and MTHFR c.677C>T genotype. Possible confounders were included in the linear regression model when they had a p-value <0.20 in univariable analysis. Possible confounders that were entered into the linear regression model were considered significant when they changed the β > 10%. A Pearson correlation coefficient was calculated to assess the association between erythrocyte non-methyl THF / erythrocyte 5 methyl THF / erythrocyte sum folate levels at T0 and T1 and erythrocyte MTX-PG_1-5_ (and subdivided in MTX-PG_1-3_ and MTX-PG_4-5_) at T1.

## Supporting information

S1 Fig Treatment protocol M—DCOG ALL10 protocol. (TIF)

S1 File Dataset. (CSV)

## Abbreviations

ALL: Acute Lymphoblastic Leukemia
DNA: Deoxyribonucleic Acid
EDTA: Ethylenediamine Tetraacetic Acid
HD-MTX: High-Dose Methotrexate
MEC: Medical Ethical Committee
MTHFR: Methylenetetrahydrofolate Reductase
RNA: Ribonucleic Acid
THF: Tetrahydrofolate
