# Supplementary figures and images for: Changes in intracellular folate metabolism during high-dose methotrexate and Leucovorin rescue therapy in children with acute lymphoblastic leukemia
Source: PLoS One. 2019 Sep 17;14(9):e0221591. doi: 10.1371/journal.pone.0221591 (PMC6748431; doi:10.1371/journal.pone.0221591)

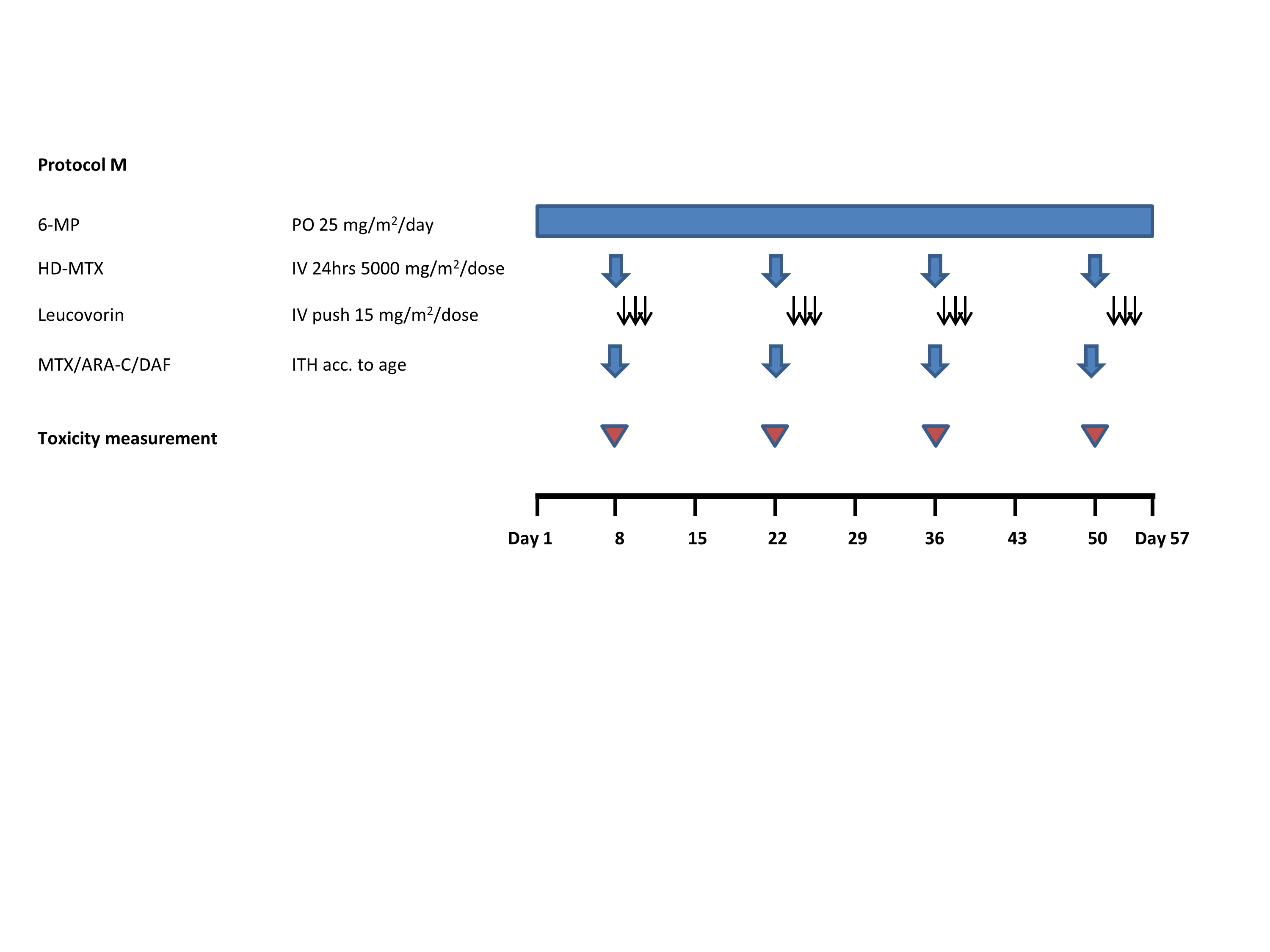

Supplement: S1 Fig — (TIF) [file pone.0221591.s001.tif]
